# Oxidative Stress and Antioxidant Defense Mechanisms in *Sepia esculenta* Larvae Induced by Co-Exposure to Environmental Cadmium and Copper

**DOI:** 10.3390/antiox15060695

**Published:** 2026-05-30

**Authors:** Xueyu Zhu, Xiaokai Bao, Mingzhi Guo, Weijun Wang, Xiumei Liu, Jianmin Yang, Zan Li

**Affiliations:** 1 School of Fisheries, Ludong University, Yantai 264025, China; 2024120925@m.ldu.edu.cn (X.Z.); 11230611062@stu.ouc.edu.cn (X.B.); 2025120930@m.ldu.edu.cn (M.G.); wangweijun@ldu.edu.cn (W.W.); yangjianmin@ldu.edu.cn (J.Y.); 2 College of Life Sciences, Yantai University, Yantai 264005, China; liuxiumei@ytu.edu.cn; 3 College of Marine Life Science, Ocean University of China, Qingdao 266003, China

**Keywords:** cadmium exposure, copper exposure, heavy metals, synergistic effects, *Sepia esculenta*, protein–protein interaction network, oxidative stress

## Abstract

Cu and Cd, as common heavy metals occurring in the oceans, can induce oxidative stress and toxic responses in marine organisms. Important economic cephalopods inhabit the eastern coastal areas of China, and exposure to Cu and Cd poses a threat to their normal physiological activities, resulting in serious inhibition of their growth. However, the underlying toxicological mechanisms affecting these cephalopods’ larval stages remain to be elucidated. Here, indicators of oxidative stress and transcriptomics were employed to analyze the toxicological mechanisms of *S. esculenta* larvae exposed to Cd and Cu. GO and KEGG analysis results indicated that material transport, cellular processes, DNA replication, and other processes were inhibited. A comprehensive analysis of a protein–protein interaction network and KEGG pathways was used to explore the mechanism underlying the toxicity of co-exposure to Cu and Cd toward *S. esculenta* larvae. We found that Cu and Cd induce significant damage and oxidative stress. The results showed that among 20 identified key genes, ITGA4, LAMA1, and LAMC1, which are involved in the adhesion and connection between cells and the extracellular matrix; COL6A1, COL6A3, COL6A4, and COL6A6, which maintain the integrity of the extracellular matrix; and ABCA1, ABCC5, and ABCC7, which regulate the transmembrane transport of Cu and Cd were involved in the mechanism of toxicity. We suggest that co-exposure to the metals primarily inhibits the connection and adhesion between the cells of the larvae and disrupts the structure and function of the extracellular matrix. The results provide a foundation for understanding the toxicological mechanism of *S. esculenta* and should be of benefit to artificial breeding programs.

## 1. Introduction

In recent years, rapid industrial development has led to the discharge of substantial quantities of polluting heavy metals, along with other common pollutants, into the marine environment. These can gradually accumulate in the bodies of marine organisms, eventually leading to growth inhibition and behavioral disorders [1,2,3,4,5,6,7,8,9,10,11]. Heavy metals in the oceans are diverse, with copper (Cu) and cadmium (Cd) being the most common and highly concentrated pollutants [12,13,14]. Both metals have high rates of bioaccumulation and degrade slowly, and both can inhibit growth, swimming, predation, and other life processes when the accumulated amounts exceed the tolerance thresholds of organisms [15,16,17,18,19]. Similarly, Cd and Cu can induce oxidative stress, destroying the structure of biological tissues and organs and thereby suppressing immune and metabolic processes [20,21,22,23,24,25].

*Sepia esculenta*, a species of mollusk occurring along the eastern coast of China, is an economically important cephalopod [26,27,28,29,30,31]. It is more vulnerable as a larva, and harsh environments can significantly affect its development compared to other marine organisms [32,33]. In this species, heavy metals can lead to reduced hatchability, oxidative damage, and the death of the larvae [34]. Therefore, further investigations are needed concerning the toxicological mechanisms of combined heavy metal exposure toward *S. esculenta* larvae.

As an important method in biological analyses, RNA-Seq can characterize biological phenomena at the molecular level [35,36,37]. The method has been widely used in studies of model organisms and has recently been applied to non-model organisms [38,39,40,41]. In recent years, RNA-Seq has been used in studies of the molecular mechanisms underlying damage from heavy metal exposure in marine organisms. For example, Pan et al. [42] and Gao et al. [43] found that Cu inhibited lipid metabolism and exhibited metabolic toxicity toward *Danio rerio*. Similarly, Cr (VI) has been shown to induce oxidative stress in *Oryzias melastigma* and inhibit protein synthesis, energy metabolism, the immune response [44]. Hence, we applied RNA-Seq to explore the results of exposure of *S. esculenta* larvae to heavy metals.

In this study, MDA, GST, SOD, and MT were used to evaluate the levels of oxidation of *S. esculenta*. Functional enrichment was then employed to identify key terms and signaling pathways. The key signaling pathways identified by a Kyoto Encyclopedia of Genes and Genomes (KEGG) enrichment analysis and protein–protein interaction (PPI) networks were employed to elucidate the toxicological mechanisms underlying acute co-exposure to copper and cadmium in *S. esculenta* larvae. This study investigated the mechanism of acute stress in *S. esculenta* larvae exposed to a combination of heavy metals. Previous studies have explored the defense mechanisms of cephalopods co-exposed to heavy metals using experimental methods [45,46,47]. The findings will give researchers deeper insight into the responses of invertebrates under heavy metal-induced toxic stress. This knowledge is significant for the development of artificial breeding of marine mollusks.

## 2. Materials and Methods

### 2.1. Exposure Experiment

In the research, we collected adult *S. esculenta* (weight: 348.93 ± 11.28 g, mantle length: 13.79 ± 0.20 mm) from the sea area near Qingdao and raised in a factory aquaculture pond for 1 week to ensure that the adult body adapts to the environment. Eggs were collected daily from the pond using an attachment net and transferred to a perforated plastic basin. Following collection, the basin was moved to a dedicated acclimation pond equipped with continuous flow-through seawater exchange and supplemental aeration. This experiment used larvae that had just completed hatching (about 28 days). The larvae were assigned to two groups, a control (C) group and a co-exposure (CuCd) group. Larvae were randomly allocated to the control and co-exposure groups using a simple random sampling method. By consulting a large number of references on the concentration of heavy metals in aquaculture areas, including both non-polluted areas and polluted areas [48,49], and referring to the concentration of Cu and Cd exposed in previous mollusk studies [17,49,50,51]. Finally, we set the concentration of Cu and Cd to 50 μg/L. Thus, the control group was raised in natural seawater, while the water for the experimental group contained 50 µg/L Cu and Cd. The physicochemical conditions of the seawater during the exposure period were maintained as follows: temperature, 21 °C; salinity, 30 ± 0.5 ppt; pH, 8.1; and dissolved oxygen, 5.7 mg/L (real-time monitoring by portable multi-parameter water quality detector). Each group comprised 100 larvae with approximately equal hatching times. In view of the vulnerability of the *S. esculenta* larvae, we referred to the current research on the short-term acute exposure of mollusks to heavy metals [51,52]. Therefore, in this study, we exposed the *S. esculenta* larvae to Cu and Cd for 4 h and 24 h. Samples were taken at the beginning of the experiment at 0 h (C_0h), followed by sampling at 4 h (C_4h and CuCd_4h) and 24 h (C_24h and CuCd_24h). Immediately after completion of the sampling, the larvae were frozen using liquid nitrogen and placed in cryopreservation tubes. The sample size of 100 larvae per group was chosen based on the feasibility of the experimental setup and the capacity of the rearing system. For RNA-sequencing, three biological replicates per group were used, with each replicate consisting of three randomly selected larvae pooled together. This pooling strategy was adopted to minimize individual biological variation while maintaining sufficient RNA yield for library construction.

### 2.2. Biochemical Indicator Testing

Nine juvenile *S. esculenta* were randomly selected and mixed in groups of three to obtain samples, and the samples, after grinding and mixing, were used to obtain the tissue fluid to be tested. Detection of GST, SOD, MDA, and MT activities and protein concentrations of samples was performed using commercial kits (Nanjing Jiancheng Bioengineering Institute, Nanjing, China; Shanghai Enzyme-linked Biotechnology, Shanghai, China). Specific methods can be found in Marklund and Marklund [53], Eren [54], Li [55], and Yang, et al. [56].

### 2.3. RNA Extraction and Sequencing

The TRIzol method was employed for total RNA extraction, following the manufacturer’s recommendations [57]. Assessment of RNA quality was carried out using an Agilent 2100 bioanalyzer (Agilent, Santa Clara, CA, USA) [58]. In each group, nine intact larvae were selected at random and pooled to form three replicates (a replicate comprised three samples). The mixing was based on the molar mass to form biological repetitive sequences. A library was constructed using the three replicates [59]. An Illumina NovaSeq 6000 (Illumina, San Diego, CA, USA) was employed for sequencing to obtain raw reads, and clean reads were obtained after quality control and filtration. HISAT2 (v2.0.5) was employed to map the clean reads to the reference genome.

### 2.4. Screening and Functional Characterization of DEGs

In this research, DESeq2 was employed to identify DEGs using the following criteria: *p*-value ≤ 0.01 and fold change ≥ 1 [60]. Functional enrichment analysis for key terms and signaling pathways was performed with the DAVID program [61].

### 2.5. PPI Construction and Validation of DEGs

Construction of a PPI network was carried out using STRING, with the inclusion of DEGs significantly enriched in key signaling pathways [62]. DEGs significantly enriched in key signaling pathways were used for the construction of the PPI network. Based on the combined results of the KEGG and PPI analyses, key DEGs (17 genes with protein interactions or KEGG pathway participation) and hub DEGs (three genes with the largest number of protein interactions or KEGG pathway participation) involved in the biological process category were identified and validated by qRT-PCR [63]. Specific primer sequences are provided in by Table S1.

### 2.6. Statistical Analysis

All biochemical assays were performed in triplicate. Data are presented as mean ± standard deviation (SD). Differences between groups were analyzed by the Kruskal–Wallis test and then analyzed by Dunn’s multiple comparison test. A significance threshold of *p* < 0.05 was applied. For transcriptomic data, differential expression analysis was performed using DESeq2 (version 1.30.0), with the criteria |log_2_(fold change)| ≥ 1 and *p*-value ≤ 0.01.

## 3. Results

### 3.1. Biochemical Indicators of Combined Exposure

**Figure 1:**
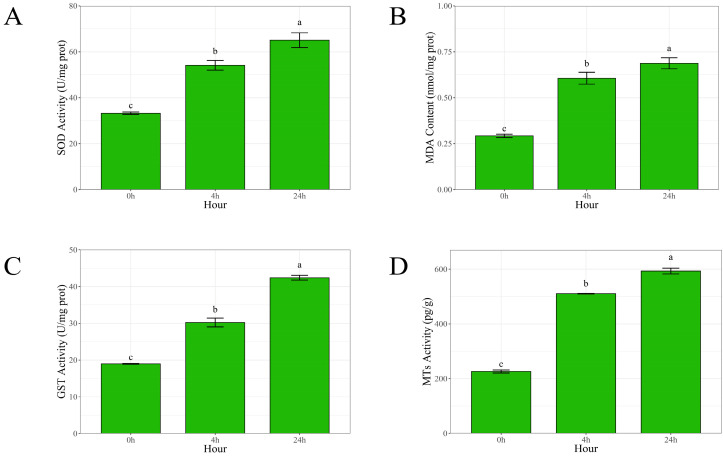
(**A**) SOD content, (**B**) MDA content, (**C**) GST content, and (**D**) MT content. Bar graphs labeled a, b, and c indicate significant differences.

Exposure time was positively correlated with SOD, MDA, GST, and MT contents (Figure 1).

### 3.2. Transcriptome Sequencing and Mapping

Sequencing produced an average of 44,189,683 raw reads per sample, resulting in 43,663,517 clean reads after quality filtering. The average Q20 and Q30 scores were, respectively, 97.44% and 93.02%, and the mean GC content of clean reads was 39.47% (Table S2).

### 3.3. DEGs

**Figure 2:**
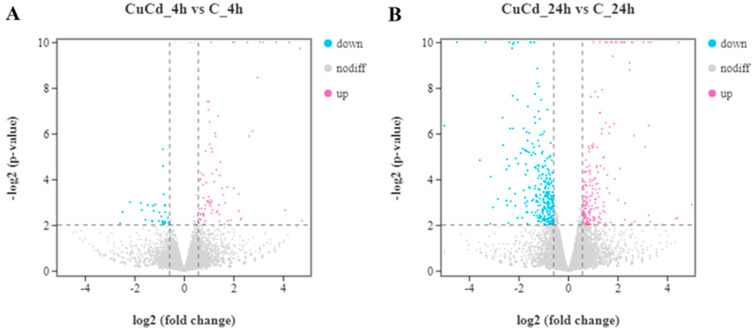
Volcano map of DEGs. (**A**) Distribution of DEG expression levels at 4 h of exposure. Each dot represents a gene. (**B**) Distribution of DEG expression levels at 24 h of exposure.

**Figure 3:**
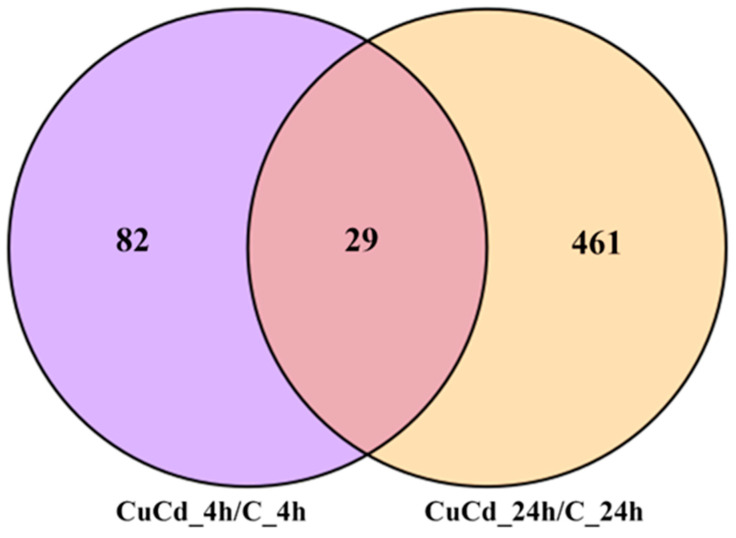
Venn diagram of the distribution of DEGs. Among them, the intersection of 29 DEGs may play an important role in the combined exposure of Cu and Cd at different times, and may be the key genes regulating oxidative stress.

**Figure 4:**
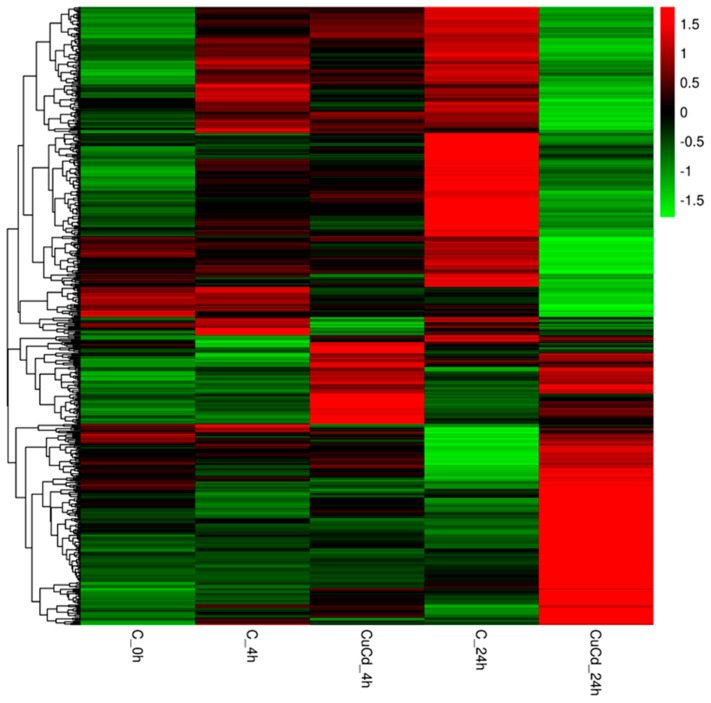
Clustering heatmap of DEGs. Each row represents a gene; each column stands for a group.

At 4 h of exposure, 81 upregulated and 30 downregulated DEGs were identified; further, 206 and 284 DEGs were upregulated and downregulated at 24 h of exposure, respectively (Figure 2). In addition, 572 DEGs were identified within 24 h of exposure, and 29 of those DEGs were identified at both time points (Figure 3). The expression patterns of DEGs are shown in a heatmap (Figure 4).

### 3.4. Enrichment Analysis of DEGs

**Figure 5:**
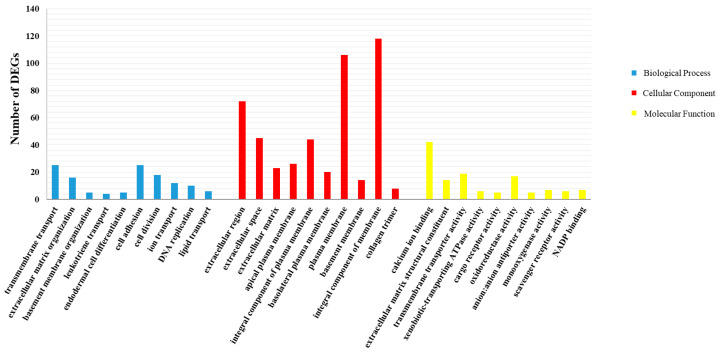
Top 10 significant GO terms. Among them, blue represents biological process, red represents cellular component, and yellow represents molecular function.

**Figure 6:**
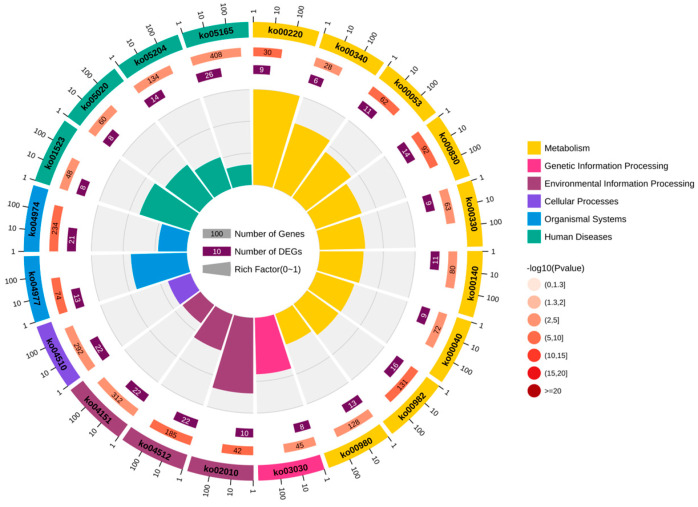
Top 20 level-2 KEGG signaling pathway results. Among them, different colors represent KEGG signaling pathways at different Level-2 levels.

A total of 114 key GO terms were identified through the analysis (Figure 5). Among these, terms like transmembrane transport and cell adhesion are involved in regulating oxidative stress processes. KEGG pathway analysis revealed enrichment in pathways associated with cell growth and death, and signal transduction, both of which regulate oxidative and toxic responses (Figure 6). The enrichment of pathways such as the PI3K-Akt signaling pathway, the neuroactive ligand–receptor interaction signaling pathway, and the MAPK signaling pathway suggests that Cu and Cd co-exposure may induce significant larval oxidative stress (Table 1).

### 3.5. Hub DEG Identification and Verification

**Figure 7:**
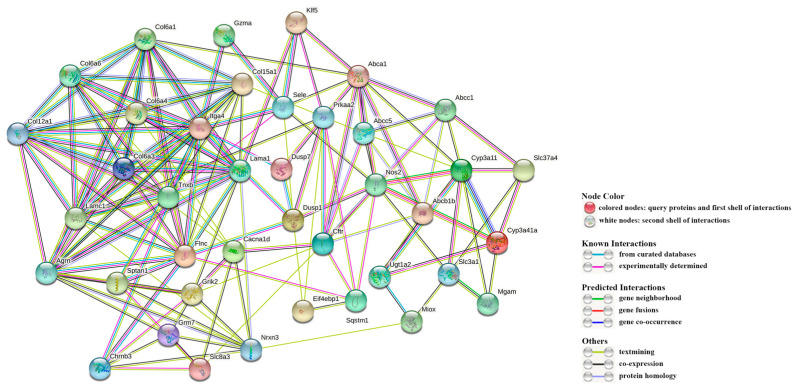
PPI network. The colored circles represent proteins, and the connecting lines represent interactions.

**Figure 8:**
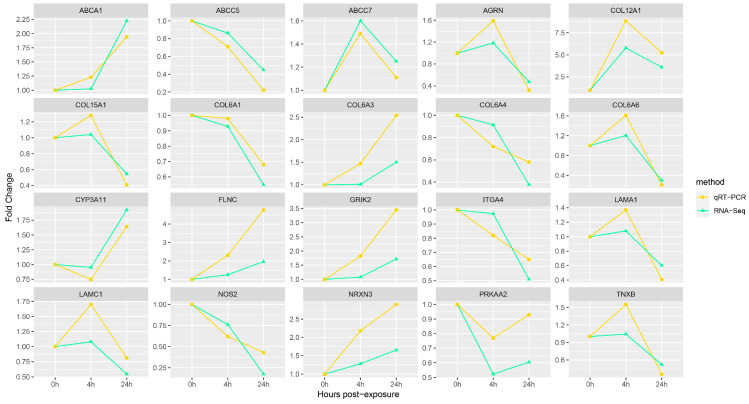
Results of qRT-PCR. Lines with different colors represent the results of qRT-PCR and the RNA-Seq method. Among them, the green lines represent the results of RNA-Seq, and the yellow lines represent the results of qRT-PCR.

A PPI network was constructed using the 41 DEGs enriched in the pathways listed in Table 1 (Figure 7). Table 2 lists the resulting network parameters. Based on the number of protein interactions, the DEGs ITGA4, LAMA1, and LAMC1 were identified as hub genes, and 17 DEGs were identified as key genes (Table S3). The RNA-Seq results were validated by the consistent expression trends demonstrated via qRT-PCR (Figure 8).

## 4. Discussion

### 4.1. Analysis of Toxicity Indicators

Heavy metals cause significant changes in ROS concentrations in organisms [64,65]. ROS at normal concentrations is involved in key processes in the life activities of organisms, such as regulating cell differentiation and participating in immune signaling [66,67]. Tissue damage, oxidative damage, DNA strand breaks, and other abnormalities will occur when the concentration of ROS exceeds the normal level for the organism [68,69]. Organisms produce large amounts of antioxidants such as SOD and MDA to activate the antioxidant defense system and mitigate the adverse influences of oxidative damage [70,71]. SOD is a key antioxidant in organisms. Superoxide anion radicals can be catalyzed by SOD to produce H_2_O_2_, which is subsequently decomposed into water and oxygen [72,73]. MDA, as a major product of polyunsaturated fatty acid peroxidation, can indicate the level of lipid peroxidation in organisms [74,75]. The findings of the research demonstrated significant increases in the activities of SOD and MDA after co-exposure, suggesting that heavy metals caused oxidative damage to the tissues of the larvae and impaired cell membrane integrity. Toxicity-associated enzymes are also capable of indicating the toxicity of heavy metals. GST, an enzyme that regulates cellular detoxification, modulates the degree of binding between GSH and heavy metals to mitigate the damage produced by the metals [76,77]. MT can bind to metals to produce MT complexes that in turn reduce the amount of heavy metals in the cells, and it can also bind to other proteins that already bind metal ions to reduce tissue damage caused by heavy metals [78,79]. The significant increase in two metal toxicity indicators, GST and MT, indicated that co-exposure significantly affected the *S. esculenta* larvae. The significant increases in various biomarkers in the larvae demonstrated that heavy metals significantly induced substantial oxidative stress and that their antioxidant system upregulated the expression of relevant antioxidant enzymes to mitigate the negative effects of co-exposure.

### 4.2. GO Functional Enrichment Analysis

The GO enrichment results showed that there was significant inhibition after co-exposure in processes such as ion transport and cell division. The enrichment of the ion transport term indicates that co-exposure may hinder ion transport and destroy the ion homeostasis of *S. esculenta* larvae [80,81]. DEGs involved in DNA replication and cell division were significantly downregulated, suggesting damage to cell structure and function [82,83]. Meanwhile, transmembrane transport and bicarbonate transport were significantly enriched, indicating that the material transport function of *S. esculenta* larvae may have been affected, and the metabolism of the larvae may have been inhibited [84,85]. The enrichment of extracellular matrix and basement membrane organization terms may reflect tissue damage induced by co-exposure in the larvae, as suggested by the significant upregulation of SOD and MDA activities [86,87].

### 4.3. Cell Connectivity and Extracellular Matrix Dysfunction

The results of SOD, MDA, and function enrichment analyses showed that co-exposure may significantly induce oxidative damage to the extracellular matrix (ECM), destroying its structure and inhibiting its function. Moreover, heavy metal exposure can affect the connection and adhesion between the ECM and cells and induce cell–matrix separation.

#### 4.3.1. Destruction of the Structural Integrity of the ECM

As a highly dynamic three-dimensional macromolecular network of non-cellular origin, the extracellular matrix is formed by collagen, laminin, fibronectin, and a range of other glycoproteins. It is primarily distributed within the connective tissue, and it is essential in maintaining the stability of tissue structures and cell functions [88,89]. The ECM modulates cellular functions—including proliferation, adhesion, growth, and migration—in most tissues via its engagement with cell surface receptors [89,90]. In connective tissues such as muscles, the ECM regulates signal transduction, growth factor expression, and proliferation and differentiation of muscle stem cells. Disorders of the ECM induce muscle remodeling, fibrosis disorders, and inhibition of muscle function, thus significantly increasing mortality rates [91]. In this study, most of the genes associated with the ECM–receptor interaction signaling pathway and ECM organization terms were significantly downregulated, indicating that ECM functions such as signal transduction and cell adhesion may have been inhibited, and the structure of the ECM may have been seriously damaged.

Collagen (COL) is the most abundant protein in tissues, and it is synthesized and differentiated by fibroblasts in the ECM [92]. As a natural tissue scaffold material, collagen plays a significant part in the maintenance of the ECM’s function and structure [93]. Meanwhile, COL is implicated in processes like cell signal transduction, cell adhesion, cell proliferation, and material transmembrane transport to maintain tissue development and growth [92,94]. Beaded-filament-forming collagen (COLVI) is a COL subfamily. It is an important component of the ECM, forming microfibers in the ECM and several superstructure components [94]. In addition, it engages in interactions with other ECM proteins, including hyaluronic acid, proteoglycan, and COLIV, in the basement membrane (BM) to jointly regulate the growth and differentiation of tissues [90]. Moreover, CON functions in the regulation of cell migration, signal transduction, adhesion, and other processes and in sustaining the functional completeness of connective tissues [95,96,97]. In this study, four COLVI subfamily genes, COL6A1, COL6A3, COL6A4, and COL6A6, were differentially expressed after metal co-exposure and were significantly enriched in the ECM–receptor interaction signaling pathway and ECM organization terms. These genes are speculated to serve critical functions in the oxidative damage to the ECM. Previous findings have revealed that COL6A1 can regulate the interaction between the BM and other ECM components to promote transmembrane transport and signal transduction while maintaining the integrity of the BM to maintain tissue stability [94,98,99,100]. COL6A3 interacts with other molecules in the ECM to regulate the formation of microfibers, and it functions in maintaining the stability of the BM [101,102]. COL6A4 and COL6A6 regulate the growth, development, and differentiation of newly formed tissues by regulating cell adhesion and migration [94,103,104]. COL6A3 was significantly upregulated, and the other three genes were significantly downregulated within 24 h of co-exposure. According to earlier studies, reduced expression of COLVI subfamily genes can induce muscle sclerosis and atrophy [94,105,106]. We preliminarily speculate that Cu and Cd may induce connective tissue damage and inhibit muscle function, thereby affecting important life activities such as respiration and swimming of *S. esculenta* larvae and inhibiting their development and growth. The observed significant increases in SOD and MDA activities support this hypothesis. Previous findings have revealed that increased COL6A3 expression can reduce the survival rates of organisms [96], consistent with the results of this study, but larval death occurred in the experiment, and the specific reasons need to be explored in subsequent experiments.

#### 4.3.2. Impaired Cell–Matrix Adhesion Function

Cell–matrix adhesion has significant functions in maintaining the stability of cellular development, survival, migration, and other processes. Impaired adhesion functioning can induce immune disorders, metabolic disorders, organ development disorders, and other pathological processes [107,108,109,110]. The molecules that regulate the adhesion process between cells and the ECM include laminin, integrin, and focal adhesion. Laminin is an important adhesion molecule that connects other components of the ECM with transmembrane proteins on the cell membrane through cell adhesion and regulates signal transduction and material transport between cells and the ECM [111,112]. LAMA1 and LAMC1 are laminins that exist on the BM, and they play key roles in the early tissue and organ development of larvae, regulating cell differentiation, adhesion, and migration and thereby maintaining tissue development for survival [90,113]. Their abnormal expression can inhibit the cell adhesion process and induce multiple biological dysfunctions, such as muscular dystrophy, blurred vision, and nervous system disorders [113,114,115]. In this study, the expression of LAMA1 and LAMC1—identified as hub genes—was significantly downregulated in the larvae of *S. esculenta* after co-exposure, implying that Cu and Cd may significantly inhibit the cell adhesion process, destroy the integrity of the BM, and inhibit the functional interaction between the BM and other molecules in the ECM. Integrin is a protein connecting the BM and the cytoskeleton to maintain the development of tissues and cells [116]. Meanwhile, integrin-mediated cell adhesion affects the survival and proliferation of cells and signal transduction [117]. As a key adhesion factor in the integrin family, ITGA4 is involved in connecting the cytoskeleton and ECM and in regulating the signal transduction and adhesion process between cells and the ECM [90,118]. Significant downregulation was observed for ITGA4, another hub gene identified in this study, upon co-exposure. We speculate that co-exposure may inhibit cell adhesion and thus affect cell–matrix functional interactions, reducing cell adhesion and signal transduction within the ECM and possibly inducing cell–ECM separation, significantly affecting the growth and development of the body of *S. esculenta* larvae. Importantly, the significant enrichment of the cell adhesion molecule signaling pathway and cell adhesion terms supports this conjecture. It is worth noting that the integrin family generally involves multiple gene interactions to jointly regulate the molecular functions of organisms. In this study, only ITGA4 was identified as the key gene regulating toxicological processes. Therefore, we hypothesize that ITGA4 may be a specific integrin family gene regulating cell–matrix adhesion of co-exposed *S. esculenta* larvae. Focal adhesion plays a key role in regulating the adhesion between the cytoskeleton and the ECM [119,120] by mediating integrin and promoting the transport of materials and signal transduction between cells and the ECM [121,122]. In the research, the focal adhesion signaling pathway was significantly enriched and downregulated, indicating that cell–matrix adhesion was inhibited and cell functions were destroyed in co-exposed *S. esculenta* larvae. The downregulation of LAMA1, LAMC1, and ITGA4 and the inhibition of focal adhesion signal are closely related to the observed increase in oxidative stress. Integrin-mediated adhesion loss disrupts the survival signaling pathway that maintains the expression of antioxidant enzymes, resulting in increased SOD, MDA, GST, and MT levels. The significant enrichment of cell adhesion molecule signaling pathway supports this explanation. The combined exposure of Cu and Cd destroyed the cell–matrix adhesion of *S. esculenta* larvae, leading to cell–ECM separation and further causing oxidative stress damage. Thus, we found that after co-exposure, the cell–matrix adhesion functions of the larvae may have been severely damaged; the cells may have lost contact with the ECM, and the cell function may have been disordered.

#### 4.3.3. Detoxification Process Mediated by ABC Transporters

Organisms activate a series of detoxification processes after heavy metal exposure. In our research, the ABC transporter signaling pathway was significantly enriched, indicating that ABC transporters are involved in regulating the detoxification process of larval *S. esculenta*. These proteins mediate the transmembrane transport of substances [123]. As the largest family of transporters, ABC transporters play significant roles in maintaining cell homeostasis, antigen processing, lipid transport, and other processes [124]. The proteins also perform efflux functions, transporting toxic substances to the outside. For example, Yin et al. found that ABC transporters of *D. rerio* embryos were involved in the efflux of Cd^2+^, thereby ameliorating heavy metal toxicity [125]. The results of Li et al.’s study on humans showed that ABC transporters promoted drug efflux and increased multidrug resistance [126]. In this study, the reason why ABCA1, ABCC1, and ABCC7 were upregulated may be that co-exposure induced the detoxification function of ABC transporters and exported Cu and Cd in the cells. The downregulation of ABCB1 and ABCC5 may indicate that co-exposure inhibits the transmembrane transport of some ABC transporters and increases metal toxicity. The observed changes in ABC transporter expression occurred together with elevated SOD activity, MDA content, GST, and MT levels. Downregulation of specific efflux transporters (ABCB1 and ABCC5) would reduce metal export, consistent with increased intracellular heavy metal retention and the concurrent oxidative stress response.

#### 4.3.4. Hypothetical Toxicological Effects of Cu and Cd in *S. esculenta* Larvae

**Figure 9:**
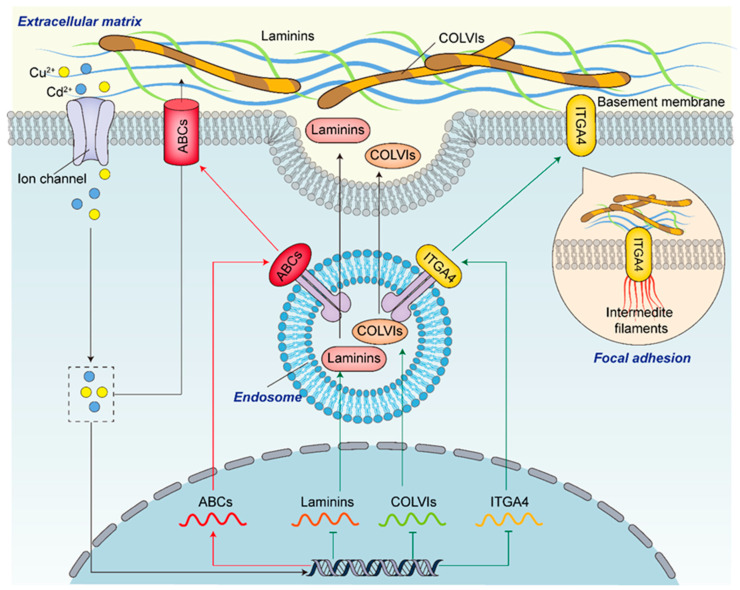
The proposed mechanism of the toxicological effects induced by co-exposure to the two metals in *S. esculenta* larvae. Under the combined stress of Cu and Cd, the integrity and normal function of the juvenile *S. esculenta* cells may be affected, and oxidative stress also occurs.

The mechanisms of toxicity associated with co-exposure to Cu and Cd in *S. esculenta* larvae have yet to be elucidated. On the basis of the present analyses and prior research, we offer preliminary speculations concerning the toxicology of Cu and Cd (Figure 9). First, Cu and Cd enter the cells of *S. esculenta* larvae through ion channels and affect the connections between cells and the ECM by inhibiting the expression of ITGA4 and the functions of focal adhesion. Meanwhile, Cu and Cd inhibit the expression of LAMA1 and LAMC1 in the BM to inhibit the adhesion of the cell membrane to the ECM and affect the expression of COLVIs, abolishing the integrity and function of the ECM. Meanwhile, ABC transporters are significantly expressed after Cu and Cd enter the cells, and these proteins promote the efflux of Cu and Cd and thereby reduce cellular damage.

## 5. Conclusions

In the research, indicators of oxidation and cellular toxicity and transcriptome analysis were used to characterize the responses of *S. esculenta* larvae after co-exposure to Cd and Cu. The significant increases in SOD, MDA, GST, and MTs suggested that the acute co-exposure significantly induced oxidative damage to the larvae. Analysis of transcriptome sequencing data revealed the inhibition of cell–matrix junctions in larvae and severe disruption of ECM structures by the heavy metals. The present research explored the toxicological mechanism of *S. esculenta* larvae after co-exposure and thus can help researchers understand the antioxidant mechanism of cephalopods, as well as provide experimental support for the captive breeding programs.

## Figures and Tables

**Table 1:** Eighteen significant KEGG signaling pathways.

Pathways	DEGs Number
ABC transporters	5
Apelin signaling pathway	3
Apoptosis	2
Ascorbate and aldarate metabolism	2
Carbohydrate digestion and absorption	2
Cell adhesion molecules	4
Cellular senescence	2
Chemical carcinogenesis—DNA adducts	3
Chemical carcinogenesis—receptor activation	5
ECM–receptor interaction	10
Focal adhesion	5
Linoleic acid metabolism	2
MAPK signaling pathway	3
Neuroactive ligand–receptor interaction	3
PI3K-Akt signaling pathway	8
Protein digestion and absorption	8
Retinol metabolism	3
Steroid hormone biosynthesis	3

**Table 2:** PPI network parameters.

Statistics	
Number of nodes	40
Number of edges	156
Average degree	7.8
Coefficient of clustering	0.494
Expected edge number	75
*p*-value	1.11 × 10^−16^

## Data Availability

The data presented in the study are deposited in the NCBI repository, under accession numbers SRR19578101, SRR19578102, SRR19578103, SRR19578104, SRR19578105, SRR19578106, SRR19578107, SRR19578113, SRR19578114, SRR20545806, SRR20545814, and SRR20545815, and are available at the following link: https://www.ncbi.nlm.nih.gov/Traces/study/?acc=PRJNA844162&o=library_name_s%3Aa (accessed on 22 May 2026).

## Supplementary Materials

The following supporting information can be downloaded at: https://www.mdpi.com/article/10.3390/antiox15060695/s1. Table S1: List of primers used for quantitative RT-PCR validation. Table S2: Sequencing quality and mapping results. Table S3: Summary of key DEGs.

## Abbreviations

The following abbreviations are used in this manuscript:

*S. esculenta*	*Sepia esculenta*
ABCA1	ATP binding cassette subfamily A member 1
ABCC5	multidrug resistance-associated protein 5
ABCC7	multidrug resistance-associated protein 7
AGRN	agrin
COL12A1	collagen type XII alpha 1 chain
COL15A1	collagen type XV alpha 1 chain
COL6A1	collagen type VI alpha 1 chain
COL6A3	collagen type VI alpha 3 chain
COL6A4	collagen type VI alpha 4 chain
COL6A6	collagen type VI alpha 6 chain
CYP3A11	cytochrome P450, family 3, subfamily a, polypeptide 11
FLNC	filamin C
GRIK2	glutamate ionotropic receptor kainate type subunit 2
ITGA4	integrin subunit alpha 4
LAMA1	laminin subunit alpha 1
LAMC1	laminin subunit gamma 1
NOS2	nitric oxide synthase 2
NRXN3	neurexin 3
PRKAA2	protein kinase AMP-activated catalytic subunit alpha 2
TNXB	tenascin XB
GO	Gene Ontology
KEGG	Kyoto Encyclopedia of Genes and Genomes
DEGs	differentially expressed genes
qRT-PCR	quantitative Reverse Transcription Polymerase Chain Reaction
PPI	protein–protein interaction
