# Supplementary material for: Oxidative Stress and Antioxidant Defense Mechanisms in Sepia esculenta Larvae Induced by Co-Exposure to Environmental Cadmium and Copper
Source: Antioxidants (Basel). 2026 May 30;15(6):695. doi: 10.3390/antiox15060695 (PMC13296053; doi:10.3390/antiox15060695)
Supplement: Supplementary file 1 [file antioxidants-15-00695-s001.zip › Table S1.pdf]

Table S1. List of primers used for quantitative RT-PCR validation.

Gene name	Forward primer (5'-3')	TM(°C)	Reverse primer (5'-3')	TM(°C)	Amplicon length (bp)
ABCA1	GAAAGCCTGAGCCGTAATC	60	AGCGTAGAAGGCCAGTAA	60	111
ABCC5	CTGGTGTCGCTGTTCTTATC	60	AACGCTCTGCAGTACTTTG	60	110
ABCC7	GATGGTCAGTGTGGAAAGAG	60	CACGTCGGAAAGTGATACAA	60	119
AGRN	GACCAGAGTTCTAGTGTTAGAG	59	CAGCATACAGAAGACGAGAA	59	123
COL12A1	AAAGCAGACCAACGCTATC	60	CGAAACTCAACACGGGATTA	60	113
COL15A1	GTCATCCAGTTGGGTGTTAG	60	CAGTGTCCGAGCAGATTATAC	60	149
COL6A1	ATGGACTTCAAGGAATACCC	59	AACCCGTACTGCCTTTATC	59	105
COL6A3	ACGGCAGAACGAACAATC	60	CACCTTTCATGTCCACTACTC	60	108
COL6A4	TGAGCAGATGATGGCATTAC	60	GCACCCTTGTTAGCATGT	60	105
COL6A6	CAGATGTTTAGGCCCAGTAG	60	CTTGTTGGGTCGCAAATTC	60	100
CYP3A11	GCAAACACTCTGTCCTTCTT	60	GGTCAGCTTCTGCACATTAT	60	129
FLNC	TGGCCGCCAAGAGTAATA	61	GCTTTCGTCACTGTCCTTAC	60	101
GRIK2	CGAACTTAGCCGCATTCTTA	60	CCTTTCAATGTCCCGTAGTG	60	103
ITGA4	GGGCGTAGCTTTGGATATAA	60	TTGTAACAGGCTCCCATAAG	59	108
LAMA1	GGAGGTGGCAAGACTTATTT	60	GAGCAGGCTTGTGGATTT	60	127
LAMC1	CCCAGGCTGGATTATTTCTT	60	TCCATACAGGCCGTAATTTG	60	110
NOS2	ATCATACGGCGACAGAGA	60	CCGCTGATAGGAGGAACTA	60	103
NRXN3	CTAACGCCTGAAGCAGATAC	60	TTAAAGCCACAAGGACACC	60	106
PRKAA2	CGGGAAGCTGAAGGATAATG	60	CATTGAGCCGGTGATCTAAT	60	134
TNXB	CAGTCAAGGCACCAAAGT	60	TCGCTTTGACGACGATTAC	60	114
