# Supplementary material for: Oxidative Stress and Antioxidant Defense Mechanisms in Sepia esculenta Larvae Induced by Co-Exposure to Environmental Cadmium and Copper
Source: Antioxidants (Basel). 2026 May 30;15(6):695. doi: 10.3390/antiox15060695 (PMC13296053; doi:10.3390/antiox15060695)
Supplement: Supplementary file 1 [file antioxidants-15-00695-s001.zip › Table S2.pdf]

Table S2. Sequencing quality and mapping results.

Samples	Raw reads	Clean reads	Q20 (%)	Q30 (%)	GC (%)	Mapping rate (%)
C_0h_1	44,822,088	44,401,358	97.42	93.02	39.89	87.71
C_0h_2	46,604,268	46,067,346	97.39	92.97	38.64	87.25
C_0h_3	42,199,716	41,745,596	97.08	92.31	39.10	86.13
C_4h_1	42,594,570	42,050,900	97.56	93.35	39.79	88.28
C_4h_2	45,122,216	44,583,624	97.37	92.89	40.01	87.80
C_4h_3	43,910,186	43,339,204	97.44	93.00	39.72	87.67
CuCd_4h_1	44,653,518	44,229,734	97.28	92.63	39.89	87.90
CuCd_4h_2	45,007,770	44,566,312	97.65	93.41	39.23	87.93
CuCd_4h_3	45,431,358	44,607,378	97.58	93.30	39.99	88.12
C_24h_1	45,374,672	44,918,056	97.51	93.17	39.82	88.32
C_24h_2	40,894,638	40,402,580	97.59	93.30	38.92	88.10
C_24h_3	42,664,646	42,060,564	97.65	93.45	38.74	87.88
CuCd_24h_1	44,072,002	43,522,418	97.31	92.83	39.85	87.85
CuCd_24h_2	45,068,272	44,699,502	97.54	93.25	38.75	87.31
CuCd_24h_3	44,425,324	43,758,176	97.21	92.48	39.71	87.75
