# Supplementary material for: Oxidative Stress and Antioxidant Defense Mechanisms in Sepia esculenta Larvae Induced by Co-Exposure to Environmental Cadmium and Copper
Source: Antioxidants (Basel). 2026 May 30;15(6):695. doi: 10.3390/antiox15060695 (PMC13296053; doi:10.3390/antiox15060695)
Supplement: Supplementary file 1 [file antioxidants-15-00695-s001.zip › Table S3.pdf]

Table S3. Summary of key DEGs.

Gene name (abbreviation)	Gene name (official full name)	Number of KEGG signaling pathways	Number of protein-protein interactions
ABCA1	ATP binding cassette subfamily A member 1	1	10
ABCC5	multidrug resistance-associated protein 5	1	7
ABCC7	multidrug resistance-associated protein 7	1	10
AGRN	agrin	1	13
COL12A1	collagen type XII alpha 1 chain	1	11
COL15A1	collagen type XV alpha 1 chain	1	12
COL6A1	collagen type VI alpha 1 chain	1	12
COL6A3	collagen type VI alpha 3 chain	4	12
COL6A4	collagen type VI alpha 4 chain	3	8
COL6A6	collagen type VI alpha 6 chain	4	9
CYP3A11	cytochrome P450, family 3, subfamily a, polypeptide 11	5	10
FLNC	filamin C	1	13
GRIK2	glutamate ionotropic receptor kainate type subunit 2	1	8
ITGA4	integrin subunit alpha 4	3	17
LAMA1	laminin subunit alpha 1	1	13
LAMC1	laminin subunit gamma 1	3	13
NOS2	nitric oxide synthase 2	1	9
NRXN3	neurexin 3	1	9
PRKAA2	protein kinase AMP-activated catalytic subunit alpha 2	2	8
TNXB	tenascin XB	3	11
